# Characterization of a Wheat Breeders’ Array suitable for high‐throughput SNP genotyping of global accessions of hexaploid bread wheat (*Triticum aestivum*)

**DOI:** 10.1111/pbi.12635

**Published:** 2016-11-23

**Authors:** Alexandra M. Allen, Mark O. Winfield, Amanda J. Burridge, Rowena C. Downie, Harriet R. Benbow, Gary L. A. Barker, Paul A. Wilkinson, Jane Coghill, Christy Waterfall, Alessandro Davassi, Geoff Scopes, Ali Pirani, Teresa Webster, Fiona Brew, Claire Bloor, Simon Griffiths, Alison R. Bentley, Mark Alda, Peter Jack, Andrew L. Phillips, Keith J. Edwards

**Affiliations:** ^1^ Life SciencesUniversity of BristolBristolUK; ^2^ The John Bingham LaboratoryNIABCambridgeUK; ^3^ Affymetrix UK LtdHigh WycombeUK; ^4^ John Innes CentreNorwichNorfolkUK; ^5^ RAGT SeedsIckletonEssexUK; ^6^ Plant Biology and Crop Science DepartmentRothamsted ResearchHarpendenUK

**Keywords:** wheat, genotyping array, single nucleotide polymorphism (SNP)

## Abstract

Targeted selection and inbreeding have resulted in a lack of genetic diversity in elite hexaploid bread wheat accessions. Reduced diversity can be a limiting factor in the breeding of high yielding varieties and crucially can mean reduced resilience in the face of changing climate and resource pressures. Recent technological advances have enabled the development of molecular markers for use in the assessment and utilization of genetic diversity in hexaploid wheat. Starting with a large collection of 819 571 previously characterized wheat markers, here we describe the identification of 35 143 single nucleotide polymorphism‐based markers, which are highly suited to the genotyping of elite hexaploid wheat accessions. To assess their suitability, the markers have been validated using a commercial high‐density Affymetrix Axiom^®^ genotyping array (the Wheat Breeders’ Array), in a high‐throughput 384 microplate configuration, to characterize a diverse global collection of wheat accessions including landraces and elite lines derived from commercial breeding communities. We demonstrate that the Wheat Breeders’ Array is also suitable for generating high‐density genetic maps of previously uncharacterized populations and for characterizing novel genetic diversity produced by mutagenesis. To facilitate the use of the array by the wheat community, the markers, the associated sequence and the genotype information have been made available through the interactive web site ‘CerealsDB’.

## Introduction

Increasing wheat yields is a major global priority for feeding the world's growing population. It has been estimated that wheat yields need to increase by 50% by 2050 to meet this demand, yet current trends are exhibiting yield plateaus (Grassini *et al*., 2013). Hexaploid bread wheat (*Triticum aestivum*) is derived from the hybridization of diploid *Aegilops tauschii* with tetraploid wild emmer, *Triticum turgidum* ssp. *dicoccoides* (Dubcovsky and Dvorak, 2007; Matsuoka, 2011; Shewry, 2009). Hybridization, domestication and strong selection pressure has reduced the level of genetic diversity available to wheat breeders, and this lack of diversity is widely recognized as a limiting factor in the breeding of high yielding varieties, particularly in response to changing biotic and abiotic stresses (Haudry *et al*., 2007; Tanksley and McCouch, 1997). The ability to assess and fully utilize the genetic diversity present in germplasm collections will inform breeding efforts, enabling potential yield increases to be attained, and it has been recognized in recent years that national efforts should be co‐ordinated to maximize progress in wheat breeding (Wheat Initiative, 2011). The ability to assess germplasm on a common genotyping platform will assist exchanges of material between countries for the introduction and mobilization of novel genetic diversity.

High‐throughput genotyping in hexaploid wheat has been made possible in recent years through the advent of next‐generation sequencing for genotyping‐by‐sequencing (GbyS; Rife *et al*., 2015) and SNP discovery (Winfield *et al*., 2012) and the subsequent development of SNP‐based marker technologies. These range from flexible, scalable single PCR‐based assays such as KASP (Allen *et al*., 2013; LGC, Herts, UK) and TaqMan^®^ (Applied Biosystems^™^, Foster City, CA) assays to high‐density fixed‐content arrays, for example the Illumina 90k iSelect array (Wang *et al*., 2014; Illumina, San Diego, CA). We recently reported the generation of an ultra‐high‐density Affymetrix Axiom^®^ array, containing 820 000 single nucleotide polymorphism (SNP) markers (Winfield *et al*., 2015). While this array represents a step change in wheat genotyping, the format is not amenable for cost‐effective high‐throughput genotyping. In addition, the majority of the markers on this array were designed to genotype polymorphisms between wheat and its near relatives and progenitors and hence are of limited direct value to wheat breeders who are specifically interested in comparing hexaploid germplasm. To overcome these limitations, we have utilized the data obtained from using the 820K wheat array in genotyping a range of diverse hexaploid accessions, to identify a set of 35 143 informative markers useful to the breeding community. To confirm the utility of the selected SNP markers, a 384 microplate format Axiom^®^ array (hereafter called the Wheat Breeders’ Array) was designed and synthesized to maximize the throughput of sample screening, including algorithms and software to enable rapid automated downstream analysis, therefore reducing required computational load.

Subsequently, we have used the Wheat Breeders’ Array to screen a large global collection of hexaploid wheat cultivar and landrace accessions. Additional germplasm screened included lines from five separate genetic mapping populations, which differ in parental material and crossing strategies, novel synthetic hexaploids and accessions subjected to mutagenesis. A diverse range of hexaploid material was included in this initial screen to allow assessment of the performance of the array SNP content in different germplasm across a range of applications of interest to wheat breeders. The design and high‐throughput nature of the Wheat Breeders’ Array makes it a potentially useful tool for research and breeding applications such as genomewide association studies (GWAS) and genomic selection. By making the array and resulting data available to the global community, we hope to demonstrate the utility of this platform for researchers worldwide. Developing global resources such as these promote rapid germplasm exchanges to boost genetic diversity and facilitate targeted breeding.

## Results

### SNP selection

SNP markers were selected from a subset of the previously described Axiom^®^ HD 820K wheat array (Table S1). Overall SNP markers were selected as described in methods to include those that were evenly spaced throughout the genome (according to genetic map position) and showed higher levels of polymorphism (measured by minor allele frequency; MAF) in the test range of hexaploid accessions, which included 108 elite hexaploid accessions of which 48 were suggested by a number of commercial wheat breeders (Winfield *et al*., 2015). Of the 35 143 SNP assays selected, 15 393 (43.8%) were considered to be co‐dominant; that is, they were able to discriminate between homozygote and heterozygote states and 19 750 (56.2%) were considered to be dominant. Of the 35 143 SNPs, 24 194 (68.8%) were transitions and 10 949 (31.2%) were transversions, compared with 72% and 28%, respectively, for the larger 820K SNP collection (Winfield *et al*., 2015).

### Genetic mapping

Five mapping populations were genotyped using the Wheat Breeders’ Array. Of the 35 143, SNP markers selected 22 001 (62.6%) were placed on one of five genetic maps (Table S3). The five different mapping populations differed in parental accessions, size of population and crossing strategy as detailed in Table 1. Two of the populations (Avalon × Cadenza and Savannah × Rialto) were generated by double haploid production from F_1_ plants, two consisted of recombinant inbred lines (RILs) generated from the F_6_ generation or F_7_ generation (Opata × Synthetic and Chinese Spring × Paragon, respectively), and one was produced by single seed descent to the F_5_ generation (Apogee × Paragon). To maximize the number of genetically mapped SNPs, a diverse selection of parental material was used to generate these populations which included spring and winter varieties, a synthetic hexaploid, the model variety Chinese Spring and a ‘super‐dwarf’, ‘rapid cycling’ cultivar (Apogee, developed for use in controlled environment experiments; Bugbee and Koerner, 1997). The number of SNPs on the array polymorphic between these specific crosses ranged from 6772 to 11 720, suggesting an average of 8793 SNPs on the array (25%) are predicted to be polymorphic between any two varieties.

**Table 1:** Mapping populations screened in this study

	Population				
	A × C	S × R	O × S	A × P	CS × P
Parent 1	Avalon	Savannah	Opata	Apogee	Chinese Spring
Parent 2	Cadenza	Rialto	Synthetic	Paragon	Paragon
Population type	Double Haploid	Double Haploid	F6‐derived RIL	F5 SSD	F7‐derived RIL
Number of individuals	128	64	60	349	269
Number of SNPs polymorphic between parents	8498	6997	9978	6772	11 720
Number of markers in genetic map	7328	6303	8820	2997	9434
Number of skeleton markers (unique position)	997	626	1509	1537	2472

Markers with greater than 20% missing data were removed prior to map construction. Of the SNPs polymorphic between the parents of the crosses, 86%, 90%, 88% and 80% of markers were able to be assigned to a linkage group on the Avalon × Cadenza, Savannah × Rialto, Opata × Synthetic and Chinese Spring × Paragon maps, respectively (Table 1). The number assigned to the Apogee × Paragon population was considerably lower (2997, 44%) due to the presence of heterozygotes in the population which complicated genotype calling at dominant SNP loci. The number of ‘skeleton markers’ initially assigned to construct the framework genetic maps was lower (626) in the Savannah × Rialto population compared to the Avalon × Cadenza population (997). This is likely to be due to the smaller number of individuals and therefore recombination events between genomic regions, and also the presence of an identical 1RS translocation on the short arm of chromosome 1B in both varieties. The Opata × Synthetic map contained 1509 skeleton markers, reflecting the greater diversity present between the parents of this cross. The Apogee × Paragon and Chinese Spring × Paragon maps had the highest number of skeleton markers (1537 and 2472) resulting from both the initial diversity present between the parental lines and the large population sizes.

**Figure 1:**
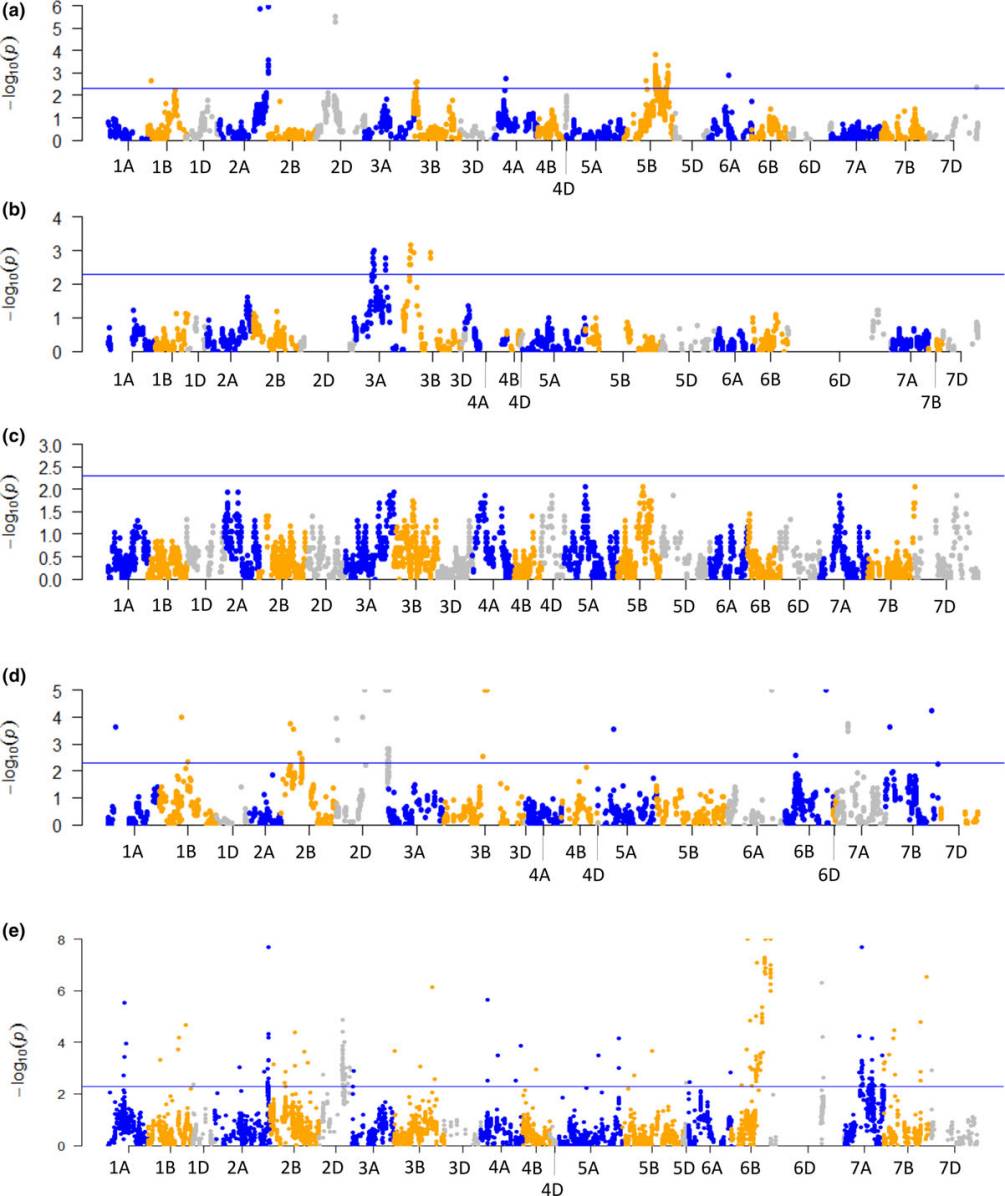
Manhattan plots showing the level of segregation distortion of SNP loci distributed across the wheat genome in four mapping populations: (a) Avalon × Cadenza; (b) Savannah × Rialto; (c) Opata × Synthetic; (d) Apogee × Paragon; (e) Chinese Spring × Paragon. The guideline indicates the significance threshold of the chi‐square test at *P* = 0.05.

The genotype assignments of SNP markers were tested for deviations from the expected 50:50 parental ratio as such markers can result in distortions in the resulting genetic maps. The distribution of segregation distortion across the genome was examined for each mapping population (Figure 1, Table S2). The population with the highest number of SNP markers exhibiting significant (*P* < 0.005) distortion of segregation was Chinese Spring × Paragon (317 SNPs), then Avalon × Cadenza (86 SNPs), Apogee × Paragon (54 SNPs) and Savannah × Rialto (38 SNPs). The Opata × Synthetic population had no SNP loci exhibiting significant distortion of segregation. The distorted loci were unevenly distributed across the genome with clusters of SNPs in specific locations (Figure 1). On the Avalon × Cadenza genetic map, significant SNPs were clustered on 8 chromosomes with the highest number on chromosome 5B (65 SNPs) in the regions 80.9, 116–130 and 157–164 cM (1, 52 and 12 SNPs, respectively). In the Savannah × Rialto genetic map, the significant SNP markers were clustered in four locations on chromosomes 3A and 3B. The clustering of significant SNPs in the Apogee × Paragon genetic map was more widespread with loci mapped to 14 locations on 10 chromosomes. The clusters with the highest number of markers were on chromosomes 2D (17 SNPs) and 3B (15 SNPs). The Chinese Spring × Paragon population had the highest number of distorted SNPs, distributed on almost every chromosome but particularly focussed in regions on chromosomes 2A (23 SNPs), 2D (97 SNPs), 6B (109 SNPs) and 7A (37 SNPs).

The markers with the most significant distortion of segregation in the Avalon × Cadenza population were mapped to chromosomes 2A (*P* = 1.12e−6) and 2D (*P* = 3.13e−6) which equates to parent1 : parent2 ratios of 31 : 83 and 77 : 29, respectively. On the Apogee × Paragon genetic map, the most distorted markers were located on chromosomes 2D (*P* = 5.17e−13), 3B (*P* = 5.38e−11), 6A (*P* = 2.0e−10) and 6B (*P* = 1.89e−6). For the Savannah × Rialto population, the most highly distorted SNP was located on chromosome 3B (*P* = 6.7e−4). The markers exhibiting the highest level of distortion on the Chinese Spring × Paragon map were located in the largest clusters of SNPs on chromosomes 2D and 6B (*P* = 2.38e−11, *P* = 7.88e−10, respectively). The direction of distortion in relation to the parental genotype in the Avalon × Cadenza population appeared biased towards Cadenza with 8 of 11 clusters and 95% SNPs being distorted in favour of the Cadenza genotype. In the Savannah × Rialto map, 24 SNPs in three locations were distorted towards Rialto, while 14 SNPs in one location were distorted towards Savannah. For the Apogee × Paragon population, 57% of the significant SNPs were distorted towards Paragon in 7 of the 14 chromosome locations. A significant bias was seen in the Chinese Spring × Paragon with 81% of SNPs in 23 locations on 16 chromosomes distorted in favour of the Chinese Spring genotype.

Markers exhibiting significant distortion of segregation in any of the populations were removed before creating the consensus genetic map. The five separate genetic maps were merged, and 21 709 markers were placed onto a consensus genetic map of all 21 chromosomes (Table 2, Table S3). The number of markers per chromosome ranged from 157 on chromosome 4D to 2168 on chromosome 2B. Overall, B genome chromosomes had the highest number of mapped polymorphisms (10 745, 48%) and D genome chromosomes had the least (2907, 13%). Individual chromosome map lengths varied from 147.2 cM (1B) to 340.2 cM (3A). The overall map length of the consensus genetic map (4645.8 cM) was higher than the DH population maps (2967.3 and 3284.1 cM) but reduced when compared to RIL‐derived population maps (4464.0–6632.3 cM).

**Table 2:** Distribution of mapped SNP loci on the Wheat Breeders array across the wheat genome

Chromosome	A x C		S x R		O x S		A x P		CS x P		Consensus	
	Number of SNPs	Length (cM)	Number of SNPs	Length (cM)	Number of SNPs	Length (cM)	Number of SNPs	Length (cM)	Number of SNPs	Length (cM)	Number of SNPs	Length (cM)
1A	425	148.1	430	178.5	457	285.1	257	262.1	558	273.9	1245	148.1
1B	956	147.3	323	122.4	759	276.5	239	291.8	795	299.4	1794	148.8
1D	292	124.6	170	71.8	103	275.2	57	171.1	226	278.4	546	238.1
2A	404	178.0	779	173.3	448	244.4	179	163.2	643	376.4	1555	180.0
2B	532	176.9	796	182.5	723	336.7	166	280.2	937	333.7	2107	187.3
2D	216	187.0	60	200.9	304	287.4	138	269.0	219	427.1	612	295.1
3A	339	184.0	375	186.6	445	345.7	168	286.9	479	340.2	1090	340.2
3B	534	179.5	487	217.4	715	313.8	212	412.1	890	344.1	1730	245.6
3D	59	129.2	24	14.7	334	248.8	12	7.7	156	401.1	465	205.0
4A	259	161.6	102	158.7	427	301.4	186	181.2	490	283.6	883	215.5
4B	304	105.0	96	51.1	336	195.7	96	186.9	273	190.2	702	152.1
4D	36	6.3	35	8.19	90	169.0	8	0.1	36	105.4	154	162.1
5A	407	218.0	551	235.4	468	382.7	166	301.6	657	429.8	1300	226.6
5B	559	191.7	305	286.55	673	318.0	194	367.8	847	404.5	1665	325.6
5D	133	126.8	148	208.2	202	347.8	0	0.0	160	349.5	416	219.6
6A	467	164.4	386	141.0	524	269.6	156	292.1	294	287.0	1060	225.3
6B	414	143.0	653	128.4	496	229.6	276	246.4	657	162.3	1509	160.9
6D	58	158.2	53	76.1	145	290.6	35	17.7	122	442.7	244	184.7
7A	395	189.1	310	139.5	477	341.8	176	253.2	546	268.1	1251	201.9
7B	348	181.8	113	56.5	543	336.7	197	281.9	345	324.5	1054	336.7
7D	105	183.6	73	129.6	151	455.8	25	191.0	104	310.4	326	248.2
Total	7242	3284.1	6274	2967.3	8820	6252.3	2943	4464	9434	6632.3	21 708	4647.4

### Array validation

The Wheat Breeders’ Array was used to screen 1843 genomic DNAs derived from 1779 unique hexaploid wheat accessions (listed in Table S4). These unique accessions included an elite collection of 505 breeding lines derived from 17 countries in Africa, Australia, the Americas, the Middle East and Europe; 436 lines from the Gediflux collection (representing Western Europe winter wheat diversity from 1920 to 1990) and 790 accessions from the Watkins global landrace collection assembled from 33 countries in the 1930s (Wingen *et al*., 2014; Burt *et al*., 2014; Miller *et al*., 2001). The unique accessions included eight synthetic hexaploid accessions and forty lines carrying various mutations or deletions in the form of cv. Chinese Spring nullisomics and monosomics (Wheat Genetic and Genomic Resources Centre, Kansas State University, USA), cv. Paragon gamma deletion lines (Wheat Genetic Improvement Network, UK) and cv. Cadenza EMS mutation lines (Table S4). The remaining samples consisted of sixty‐four replicates of named accessions sourced from different laboratories.

Genotype calls were generated as described in Experimental Procedures. Across the samples genotyped, the average call rate was 97.9%, ranging from 94.1% to 99.2% (Table S4). The accession type with the highest average call rate was mutation lines (98.1%), and the lowest was synthetic hexaploids (96.3%). The relationship between call rate and heterozygous call rate per accession was investigated. A trend was observed where samples with low call rates tended to have a higher than average het rate (a higher percentage of SNPs called AB). The DNA samples for these lines are predicted to be of lower quality as the increase in AB calls and lower call rate represents a higher number of outlier calls from the main clusters. For use as a high‐throughput genotyping platform, reproducibility is an important consideration. The call rate among duplicate samples was highly similar (ranging from 99.3% to 99.8%); however, the call rate for replicate samples prepared from the same named accessions, but from different sources, showed more variation (97.5–99.4); this is likely to reflect true within‐cultivar variation.

The total number of polymorphic SNPs was 33 326 (94.8%) of the entire array based on the screen of the collection of lines described above. A summary of the numbers of polymorphisms present unique to and shared between germplasm collections is presented in Table 3. The collections with the highest number of unique SNPs were the elite global collection (247 SNPs), the Watkins landrace collection (218 SNPs) and the synthetic hexaploid collection (144 SNPs). The collections sharing the highest number of polymorphisms were the elite cultivars, Gediflux and landrace collections, with up to 32 013 SNPs being transferrable between and useful within different collections. The lower numbers of shared polymorphisms between these and other collections (e.g. deletion and mutation lines) are representative of the narrow genetic base compromising the collections of deletion and mutation lines which are developed in a single genetic background. The effect of collection size on the number of polymorphic SNPs within a collection was also apparent (Table 4). A sharp increase in level of polymorphism was seen between collection sizes of <5 to around 50 individual accessions, reaching an average of 90% polymorphic SNPs in collections of 100 accessions.

**Table 3:** Numbers of SNPs unique to and shared between germplasm collections

	Elite cultivars	Gediflux collection	Landraces	Chinese Spring deletion lines	Paragon deletion lines	Cadenza EMS lines	Synthetic hexaploid lines
Elite cultivars	247						
Gediflux collection	31 473	43					
Landraces	32 013	31 388	218				
CS deletion lines	8822	8807	8882	65			
Paragon deletion lines	5932	5906	5913	2778	8		
Cadenza EMS lines	6603	6580	6583	2789	5312	5	
Synthetic hexaploid lines	19 266	18 662	19 035	6350	4342	46 890	144

**Table 4:** Summary statistics of cultivar collections

	Australia	Central America	Middle East	North America	North Europe	South Africa	South America	South Europe	West Europe	Gediflux
n	146	64	5	40	10	5	6	17	271	436
% P	92.5	85.3	61.2	87.4	69.5	53.8	60.0	81.1	97.3	95.2
*H*_E_	0.229	0.202	0.207	0.232	0.205	0.188	0.200	0.238	0.229	0.214
MAF	0.167	0.146	0.153	0.168	0.150	0.142	0.150	0.173	0.164	0.155
RI	1.029	0.997	1.019	1.029	0.837	0.872	1.155	1.051	0.947	0.826

n, number of samples; % P, percentage of total SNPs on the array which are polymorphic; *H*_E_, expected heterozygosity; MAF, average minor allele frequency; RI, rarity index.

**Figure 2:**
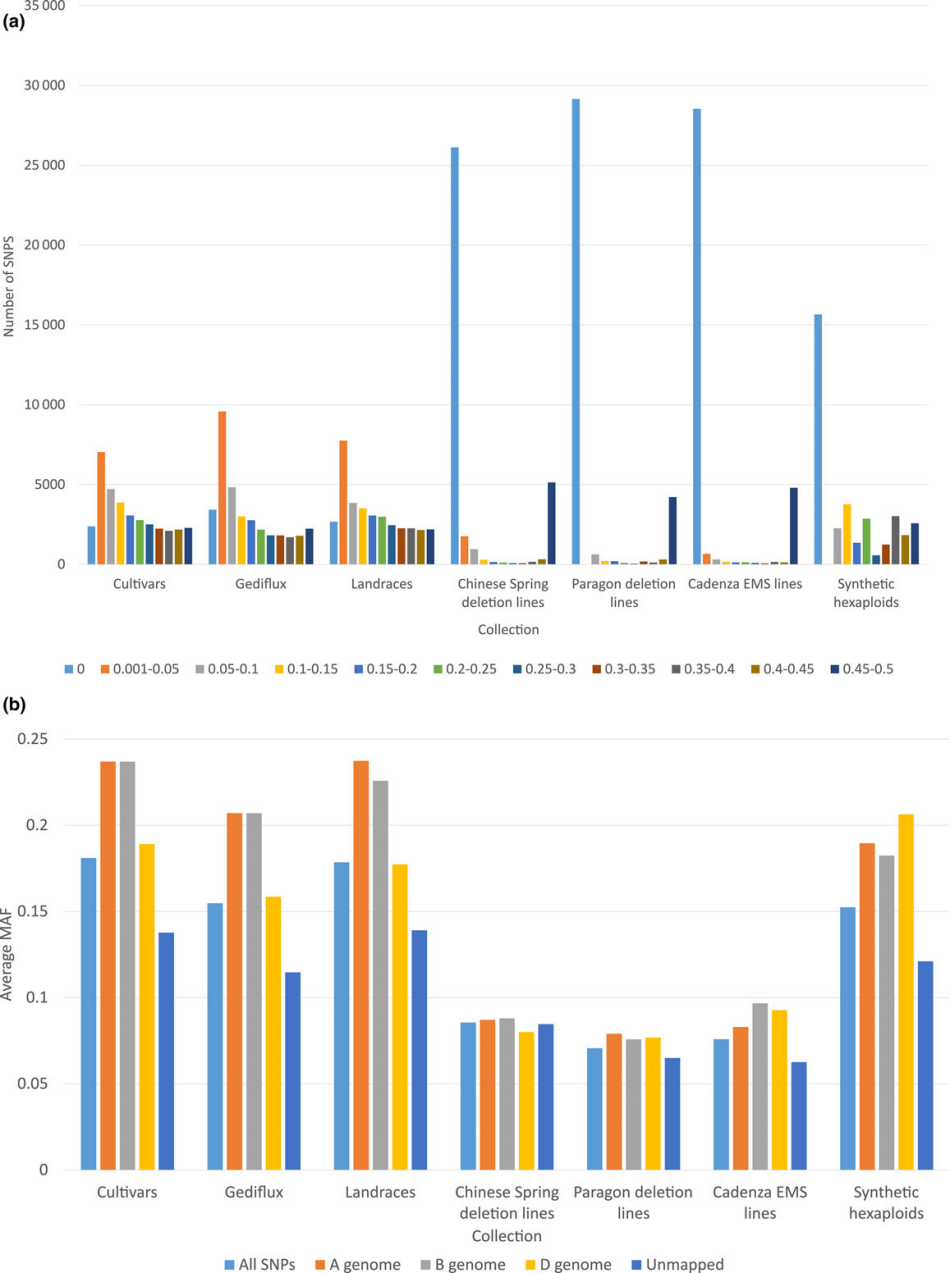
(a) Distribution of minor allele frequencies (MAFs) of SNP loci within germplasm collections. (b) Average MAF of A, B and D genome mapped SNPs in the different germplasm collections.

The minor allele frequencies of SNPs within different germplasm collections were calculated as a measure of allelic diversity (Figure 2a). The larger elite cultivars, Gediflux and landrace collections had a higher number of polymorphic SNPs, with a cumulative prevalence of intermediate to high MAF SNP loci, distributions observed previously for similar wheat collections (Wang *et al*., 2014). The MAF distributions of SNPs within the deletion and mutation lines were more skewed, with a high proportion of polymorphic SNPs showing a MAF of 0 and 0.45–0.5 (57%, Chinese Spring deletion lines; 70%, Paragon deletion lines; 73%, Cadenza EMS mutation lines). This is likely to be due to both the small sample sizes of these collections (16, 9 and 15 samples, respectively) and the limited genetic background of these collections, meaning the samples will be homozygous at most sites represented on the array. The polymorphic SNPs identified in these collections are therefore likely to be associated with deleted or mutated regions. The synthetic collection showed a bimodal MAF distribution; a high proportion of polymorphic SNP loci had either a high or low MAF, although this is partially a reflection of the small sample size of eight individuals. To further study the MAF of the synthetic lines, the average MAF of A, B and D genome mapped SNP loci was calculated (Figure 2b). The average MAF of the cultivar, Gediflux and landrace collections was very similar, with typically higher MAF observed in A and B genome markers compared to D genome markers. The average MAF of A, B and D genome markers in the deletion and mutation line collections was very similar. The synthetic collection had a higher average MAF of D genome markers compared to A and B genome markers highlighting the increase in diversity brought to the D genome by the novel *A. tauschii* accessions used in the creation of these lines.

**Figure 3:**
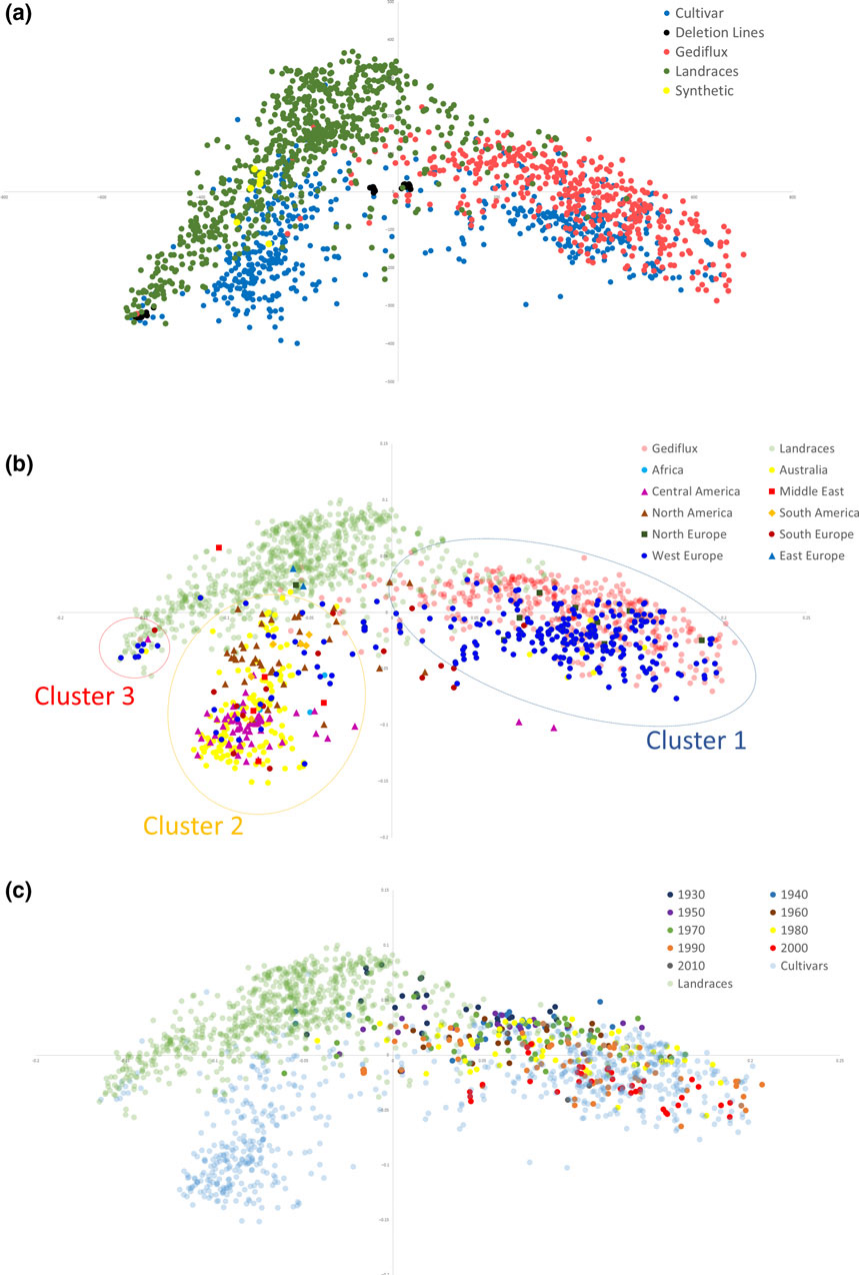
Principal coordinate analysis (PCoA) plots coloured by (a) collection, (b) country of origin (c) date of line release. Coordinate 1 is plotted along the *x*‐axis, coordinate 2 is plotted along the *y*‐axis.

The relationship between accessions was visualized by calculating a pairwise similarity matrix that was used to perform multidimensional scaling (MDS) and create principal coordinate (PCoA) plots (Figure 3). With the different germplasm collections, it was clear that accession type contributes to the structure of the PCoA plot (Figure 3a). Elite cultivars were split primarily into two clusters representing spring (negative PCo2 values) and winter (positive PCo2 values) accessions, with the winter accessions interspersed with the Gediflux collection. The Watkins landrace collection slightly overlapped in distribution with the elite cultivars but formed a distinct cluster extending towards negative PCo2 values. The deletion/mutation lines formed tight clusters representing the common genetic backgrounds to these lines. The eight novel synthetic hexaploid accessions clustered within spring cultivars and landrace accessions.

### Relationship between global elite germplasm collections

To further examine the diversity present in different elite germplasm collections, they were defined by geographical region of origin. The separate components of the collections are summarized in Table 4 including number of accessions, % of polymorphic markers, measures of diversity (HE and MAF) and rarity index (a measurement of the number of rare alleles present in each subcollection). The subcollections with the highest numbers of polymorphic markers were both from Western Europe; these were also the two largest subpopulations; the smallest subpopulations had the lowest numbers of polymorphic markers, reinforcing the relationship seen between collections size and number of polymorphic markers seen in Table 4. The subcollections with the highest genetic diversity measures were from Southern Europe and North America. The subcollection with the highest rarity index was South America (despite being one of the smallest subcollections), and the lowest was the Gediflux collection, suggesting that polymorphic alleles are widespread in this collection.

The relationship between different subpopulations was further examined by calculating the number of shared polymorphisms and genetic differentiation between subcollections (Table 5). The number of shared polymorphisms was highest between the Gediflux and Western Europe elite accessions; these also had the lowest *F*_ST_ value, which is not surprising given that they are both of Western European origin. The Western Europe and Australian collections appeared to have a high degree of similarity with considerable overlaps in polymorphic markers, although this is may also be attributed to both collections having the highest number of polymorphic SNPs overall. Overall, the majority of polymorphic SNPs were shared among populations, suggesting that there is a high transferability of SNP markers across global elite germplasm collections. High *F*_ST_ measures between populations of different geographical origin is likely to be caused by the usage of different founders or by allele frequency divergence during the development of locally adapted populations. High *F*_ST_ measures were seen in particular between Western European and Central American subpopulations. Conversely, the lowest *F*_ST_ measures were seen between Middle Eastern, Southern American and Southern Europe subpopulations suggesting use of similar founders or overlap in these breeding programmes.

**Table 5:** Number of shared polymorphisms (above diagonal) and genetic differentiation, *F*_ST_, (below diagonal) between cultivar subcollections

	Australia	Central America	Middle East	North America	North Europe	South Africa	South America	South Europe	West Europe	Gediflux
Australia		27 615	20 172	28 446	22 693	17 813	19 746	26 594	30 488	30 115
Central America	0.077		19 676	26 603	21 252	17 300	19 181	25 237	28 130	27 732
Middle East	−0.047	−0.022		19 938	16 177	14 497	16 010	19 305	20 312	20 195
North America	0.046	0.079	−0.0594		22 005	17 575	19 429	25 963	28 927	28 731
North Europe	0.141	0.185	0.023	0.101		13 757	11 009	21 085	23 057	23 027
South Africa	−0.063	0.005	−0.247	−0.051	0.043		13 536	16 970	17 879	17 718
South America	0.039	0.063	−0.134	−0.004	0.082	−0.086		18 690	19 931	19 699
South Europe	0.032	0.030	−0.116	0.009	0.044	−0.085	−0.018		26 919	26 728
West Europe	0.139	0.166	0.063	0.116	−0.033	0.069	0.111	0.068		31 371
Gediflux	0.180	0.210	0.114	0.152	−0.030	0.121	0.156	0.103	0.013	

To further examine the relationship between cultivars of different geographical origins, samples plotted by the first two principal co‐ordinates were coloured by region of origin (Figure 3b). Three main clusters were observed for elite cultivars. One cluster defined by positive PCo1 values consists of mainly Western European accessions and also includes a small number of Australian and Northern European accessions. The second largest cluster consists primarily of Australian and American accessions. Further structure is observed for this cluster with lower PCo2 values for Central American and Australian accessions and higher PCo2 values for Middle Eastern, Southern and Northern American accessions. A third small cluster consisted of accessions from Western and Southern Europe, Australia and Central America. On further investigation, the samples supplied from Western Europe are actually of Asian origin. To investigate the impact of cultivar age on genetic diversity, the date of cultivar release was used to colour accessions from Western Europe (Figure 3c). Accessions were grouped per decade, and a trend was observed with pre‐1960s accessions locating closer to the central landrace cluster and later accessions extending along the PCo1 axis, particularly in the 1980s and 1990s, suggesting an expansion of diversity during this period.

### Using the Wheat Breeders’ Array to characterize novel genetic diversity including deletions, introgressions and genomic rearrangements

The collection of lines screened on the Wheat Breeders’ Array included 40 accessions with known deletions of various types; monosomic (missing one chromosome of a pair), nullisomic (both chromosomes of a pair are deleted but substituted with those from a homoeologous genome), ditelosomic (missing part of an end of a chromosome) and gamma‐irradiated deletions (smaller deletions within a chromosome). In the case of the gamma‐irradiated lines, we were able to identify between 299 and 796 polymorphic markers compared to the Paragon control representing between 1.86% and 1.95% of the markers scored. For the five lineages examined, the majority of variable markers mapped to specific regions of which most were associated with the known deleted regions. For the Cadenza EMS mutagenized lines, for the 15 lines examined, there were between 424 and 709 polymorphic markers compared to the Cadenza control representing between 2.64% and 4.41% of the markers scored. A number of these polymorphic markers appeared to be common to all or most of the mutagenized lines, suggesting that some polymorphisms might have existed in the original stock used for mutagenesis. The breeding schedule for cv. Cadenza shows that cultivar is based on a single individual from the F_6_ generation resulting in ~3% residual heterogeneity in the final cultivar. Unique SNPs to each accession were mostly randomly distributed throughout the genome and are likely to reside in mutated regions specific to each line.

**Figure 4:**
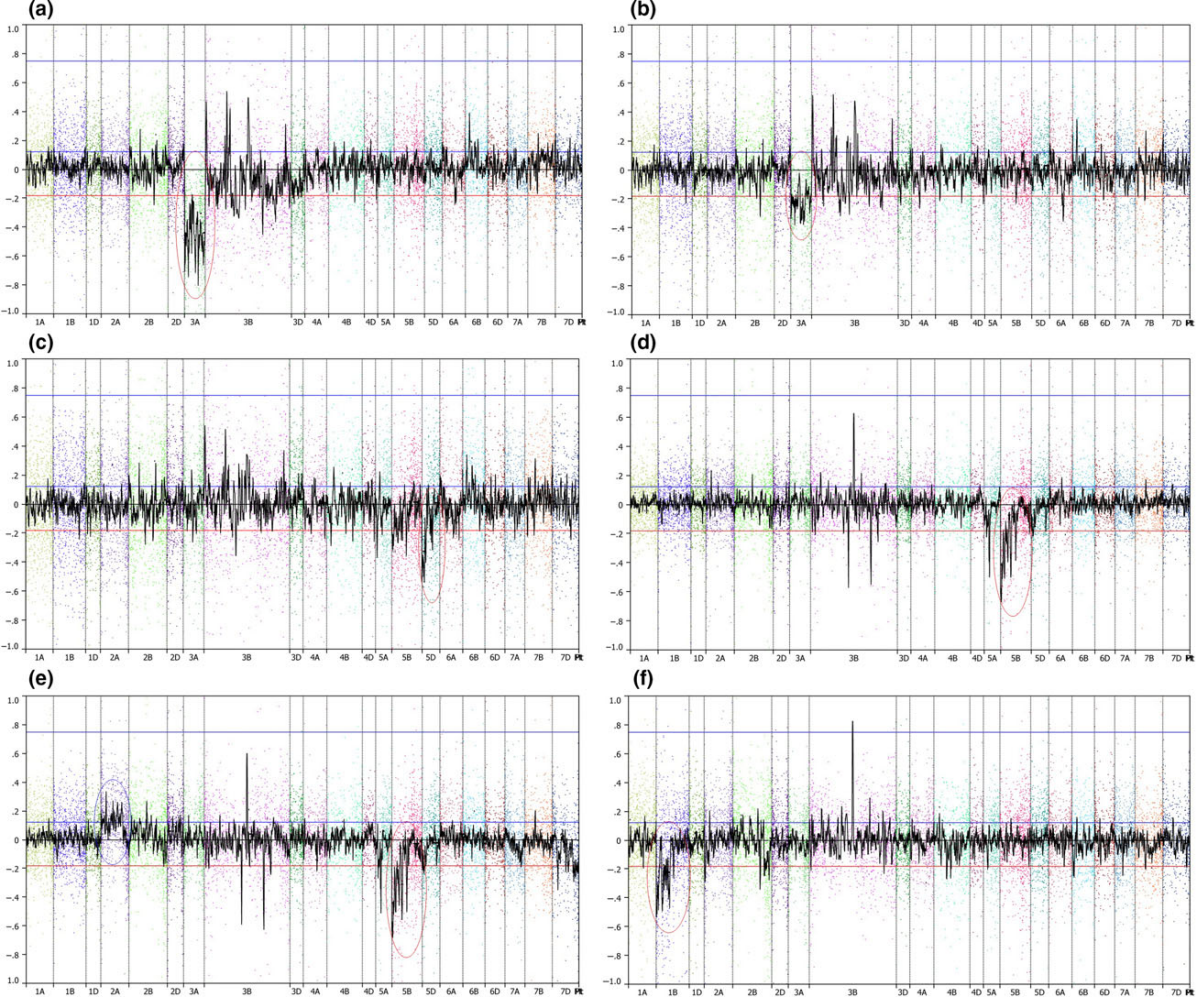
Signal intensity (Log_2_R ratio) plots of copy number variation (CNV) across the genome for different hexaploid wheat accessions. The accessions displayed are as follows: (a) Chinese Spring nullisomic 3A deletion; (b) Chinese Spring monosomic 3A deletion; (c) Chinese Spring ditelosomic 5DS deletion; (d) Paragon gamma‐irradiated 5B deletion; (e) Paragon gamma‐irradiated line exhibiting CNV loss and gain; (f) cv. Savannah, carrying the 1RS translocation from rye. Blue circles highlight copy number‐gained CNV regions, red circles highlight copy number‐loss CNV regions.

We used copy number variation (CNV) analysis to characterize the various deletion lines; using this procedure, we were able to highlight SNP loci associated with each of the deleted regions where a reduced hybridization signal was observed (Figure 4). Interestingly, evidence was also seen for an increase in signal in certain chromosome regions of some of the gamma deletion lines (4e). This apparent over‐representation implies duplication of large chromosomal segments, presumably as a result of the gamma irradiation. The same approach was applied to analyse the genotyping data from the elite cultivars. For certain accessions, regions of reduced signal were observed, associated with varieties carrying known introgressions, that is the 1RS introgression from rye (e.g. cv. Savannah, 4f). A number of additional cultivars (Keilder, Gulliver, Mercato) showed significantly low signal strength for one or more chromosomes potentially representing deletions or ancestral introgressions (Table S5).

## Discussion

We designed the Wheat Breeders’ Array to be a high‐throughput platform for the cost‐efficient generation of genetic maps between a range of parental lines and for genotyping hexaploid wheat derived from a variety of sources. To confirm the utility of the platform, we screened several mapping populations, generated from a range of hexaploid lines via different crossing strategies, to produce five high‐density genetic maps. The relatively high level of codominant SNP assays on the array enabled a map to be produced from an F_5_ population containing heterozygotes in addition to the more traditional DH populations. This may be a more cost‐effective mapping population development strategy than DHs for some purposes.

The genetic map produced using the Apogee × Paragon population revealed a high number of recombinants within the population, indicated by the high number of skeleton markers, and inclusion of this in the consensus map helped to resolve marker order in regions consigned to large ‘bins’ of markers in the other maps. Similarly, the Opata × Synthetic map increased the overall number of mapped SNPs, particularly in the D genome due to the increased diversity incorporated through use of a synthetic hexaploid as a parent of the mapping population. The lack of genetic diversity in the D genome of hexaploid wheat cultivars is a well‐documented phenomenon attributed to the genetic bottleneck experienced during the initial hybridization to create the hexaploid and the subsequent limited gene flow into bread wheat from *A. tauschii* compared to that from tetraploids into the A and B genomes (Dvorak, 2006, Halloran et al., 2008). This was also reflected in the analysis of the average MAF of A, B and D genome markers, where the synthetic hexaploid lines (bred to specifically increase D genome diversity) screened showed higher MAF in the D genome compared to the A and B genomes, a trend opposite to that observed in conventional cultivars and landraces. Using the five mapping populations, we were able to generate a consensus map consisting of 22 001 SNP markers or 63% of the total SNP markers on the array. This compares favourably to maps generated previously for similar wheat SNP arrays such as the Illumina iSelect 90k wheat array (Wang *et al*., 2014; 46 977 mapped markers, 58% of total) and the Affymetrix Axiom^®^ HD Wheat Genotyping Array (Winfield *et al*., 2015; 56 505 mapped markers, 7% of total).

The high marker density of the constructed maps highlighted features of the genome such as regions of distorted segregation which were unequally distributed across the genome. Segregation distortion of genetic loci is a potentially powerful evolutionary force that allows the enhanced transmission of a specific genetic locus (Taylor and Ingvarsson, 2003). A number of examples of significant segregation distortion were observed in the mapping populations analysed in this study. The Avalon × Cadenza population had several peaks of highly significant segregation distortion, in particular on chromosomes 2A, 2D and 5B. The bimodal peak on 5B is likely to represent the effect of the 5B–7B reciprocal translocation present in the population and donated from Avalon. This translocation is a relatively widespread chromosomal rearrangement in Western European cultivars and is thought to be of adaptive value in controlling plant growth and development. In some populations, the translocated chromosomes have been reported to be preferentially transmitted (Schlegel, 1996; Friebe and Gill, 1994). Overall, the Avalon × Cadenza population showed significant bias (95% of loci) towards inheriting the Cadenza genotype.

The Savannah × Rialto population exhibited distortion of segregation of 38 SNPs representing four loci on two homoeologous chromosomes, 3A and 3B. It is interesting to note that very similar patterns are observed on both of these homoeologous group 3 chromosomes, both having bimodal peaks which may represent genomic rearrangements (as described above) or genes of large effect. In contrast, the Apogee × Paragon population had numerous regions of distorted segregation, typically consisting of relatively small numbers of SNPs with a high level of significance, with no significant bias towards either parent. The difference in pattern of distorted loci may partially reflect how each population was produced. The DH populations were in effect ‘fixed’ at the F_1_ cross, and any regions of segregation distortion present were transferred into the DH and maintained in the population; these may have been of large effect and size. The Apogee × Paragon population has undergone further inbreeding to the F_5_ generation and multiple distorted loci of small size are observed.

Screening the Wheat Breeders’ Array with a range of hexaploid lines demonstrated its utility on a wide range of germplasm from different geographical areas and ages. Overall, a high number of polymorphisms were shared between collections, with an average of 23% of SNPs on the array predicted to be polymorphic between two random accessions. A relationship between polymorphism level and collection size was observed, with an indication that at least 30 accessions are needed to maximize the chances of fully utilizing the polymorphism content of the array. The genotyping data were further explored to examine the relationships between diverse collections of global breeding lines. In general, a high number of shared polymorphisms and low *F*_ST_ was observed between populations of different geographical origin, suggesting that there has been an overlap of germplasm used within these breeding programmes. The principal co‐ordinate plots reflect low *F*_ST_ measures with overlaps in particular between (i) Western Europe, Northern Europe and Gediflux accessions; (ii) Australian and Central American accessions; (iii) Northern American, Southern American, Southern Europe, Middle Eastern and Southern African accessions. Cluster 1 is unsurprising given the overlap between the geographical origins of these collections. Cluster 2 reflects the significant impact the CIMMYT developed lines have had on Australian breeding programmes since 1965 (Brennan and Quade, 2004). The relationships between the populations overlapping in cluster 3 are less clear, although climatic conditions within these countries are similar, making the exchange of adapted germplasm conceivable. It has been observed that during the 20th century, the global community of wheat breeders freely shared genetic materials (Kronstad, 1997), particularly in efforts led by the International Maize and Wheat Improvement Center (CIMMYT) and the International Center for Agricultural Research in the Dry Areas (ICARDA).

The hexaploid nature of the bread wheat genome means that it is amenable for both crossing with a range of wheat relatives and large‐scale mutagenesis such as gamma irradiation. As both of these procedures can increase the diversity of the hexaploid gene pool, they are becoming more widely employed by breeders and academics alike. Hence, we investigated the ability of the Wheat Breeders’ Array to characterize such material via the use of the CNV tool developed by Affymetrix (Axiom^™^ CNV Summary Tools Software v 1.1, part #600 733). By first using a collection of lines containing known deletions of different sizes and locations, we were able to characterize a range of deletions in terms of both their size and nature, that is monosomic or nullisomic. Examination of a number of lines of the variety Paragon, which had undergone gamma irradiation, allowed us to identify previous uncharacterized deletions and in addition show that a number of these irradiated lines also potentially carry duplicated regions. Finally, we used the fact that the SNP markers on the Wheat Breeders’ Array were specific for hexaploid wheat, to screen a range of hexaploid lines for the evidence of either introgressions, such as the 1RS introgression from rye, or deletions. This screen generated evidence that numerous lines probably carry deletions and introgressions, and hence, our analysis suggests that further work is needed to characterize the extent of copy number variation within the hexaploid gene pool.

The Wheat Breeders’ Array has been demonstrated to be useful for screening germplasm collections from across the globe and for characterizing sources of novel variation in a hexaploid background. As such, and given the design and high‐throughput nature of the Wheat Breeders’ Array, this tool may be applied to research and breeding approaches such as genomewide association studies (GWAS) and genomic selection. To further increase the utility of the array, we have screened five mapping populations and constructed a consensus genetic map to allocate a position to over 63% of the markers on the array. Further analysis has indicated that the markers on the array may be successfully used to identify regions of CNV and distorted segregation in the wheat genome, which in turn point towards chromosomal rearrangements and the presence of introgressions. To facilitate the use of the array by the global wheat community, the markers, the associated sequence and the genotype information have been made available through the interactive web site ‘CerealsDB’ (Wilkinson *et al*., 2012, 2016).

## Experimental procedures

### SNP selection

The original SNP collection consisted of 819 571 SNPs obtained from genic sequences derived via targeted capture re‐sequencing of numerous wheat lines and validated on the Axiom^®^ HD Wheat Genotyping Array (Winfield *et al*., 2015; Affymetrix UK Ltd, High Wycombe, UK). To select the most informative ~35 000 SNPs (the maximum permissible on the 384 Axiom^®^ genotyping platform) for inclusion on the Wheat Breeders’ Array, each SNP was assigned to an IWGSC scaffold via BLAST (Winfield *et al*., 2015). Once assigned, SNPs unique to a particular contig were selected. In cases where there was more than one SNP per contig, SNPs which had been genetically mapped on one or more of the three mapping populations used in the original analysis were selected. In cases where more than one SNP had been mapped, co‐dominant SNP markers were preferentially selected and of these the SNP marker with the highest Polymorphic Information Content (PIC) score was selected. Where no SNPs in an IWGSC contig had been mapped, one SNP was selected with co‐dominant SNPs being selected in preference to dominant SNPs and SNPs with high PIC scores being selected in preference to those with lower scores.

### Plant material

The accessions grown for DNA extraction (listed in Table S4) were grown in peat‐based soil in pots and maintained in a glasshouse at 15–25 °C with 16‐h light, 8‐h dark. Leaf tissue was harvested from 6‐week‐old plants, immediately frozen on liquid nitrogen and then stored at −20 °C prior to nucleic acid extraction. Genomic DNA was prepared from leaf tissue using a phenol–chloroform extraction method (Sambrook Il., 1989). Genomic DNA samples were treated with RNase‐A (New England Biolabs UK Ltd. Hitchin, UK), according to the manufacturer's instructions and purified using the QiaQuick PCR purification kit (QIAGEN Ltd., Manchester, UK).

### Genotyping

The Axiom^®^ Wheat Breeders’ Array was used to genotype 2713 samples (Table S4) using the Affymetrix GeneTitan^®^ system according to the procedure described by Affymetrix (Axiom^®^ 2.0 Assay for 384 samples P/N 703154 Rev. 2). Allele calling was carried out using the Affymetrix proprietary software package Axiom Analysis Suite, following the Axiom^®^ Best Practices Genotyping Workflow (http://media.affymetrix.com/support/downloads/manuals/axiom_genotyping_solution_analysis_guide.pdf).

### Genetic map construction

Individuals from five mapping populations were genotyped with the Axiom^®^ Wheat Breeders’ Array (Table 1). For each population, markers with more than 20% missing data were removed and markers were binned based on their pattern of segregation in each respective population using the ‘bound’ function in Multipoint ULD (MultiQTL Ltd., Haifa, Israel). Markers were placed into the same bin if the correlation coefficient between them was 1, and therefore, the recombination frequency between them was estimated as 0. Following binning, linkage groups were ordered and then all markers which displayed a unique pattern of segregation and did not previously fall into a bin were iteratively added into each linkage group. During this process, the inflation coefficient was set to 1.2 to ensuring that markers which caused map inflations (likely to be due to genotyping errors) were not retained.

Markers were tested for significant segregation distortion using a chi‐square test. The log10 value of the chi‐square test statistic for each marker was plotted against marker position using the R package qqman. SNP loci exhibiting significant distortion of segregation and ambiguous markers mapping to different chromosomes in different populations were removed from individual maps before creating the consensus map. The consensus map was constructed using the R package LPmerge (Endelman and Plomion, 2014). No weighting was given to the component maps.

### Dimensionality reduction

The relationship between the lines was determined by calculating a similarity matrix for all the lines. This was calculated as number of markers shared by any two lines divided by total number of markers for the two lines; markers that had missing calls for either of the lines were not used to estimate similarity. The matrices were imported into R and used to create principal coordinate plots using the classic multidimensional scaling (MDS) method, cmdscale.

### Summary statistics of germplasm collections

Summary statistics were calculated using StAMPP v1.0 (Pembleton *et al*., 2013) and the following formulae:

$$\text{Expected heterozygosity}=\text{He}=1-\Sigma \;p_{i}^{2}$$

$$\text{Rarity index}\;\left(\text{RI}\right)={\text{RI}}_{j}=\frac{1}{I}\sum_{i=1}^{I}\frac{p_{ij}}{p_{i}}$$

where *I* is the number of markers, *p*_*ij*_ is the frequency of *i*th marker in a group of cultivars *j*, and *P*_*i*_ is the frequency of *i*th marker in the total dataset.

### CNV analysis

CEL files from the Wheat Breeders’ Array were processed using the Axiom Analysis Suite, with option set to Polyploid and Inbred, with the inbred het penalty set to 4. The annotation file was generated using the Affymetrix Annotation Converter, using chromosomal locations for SNPs downloaded from the IWGSCv1 assembly on Ensembl Plants. CNV analyses were visualized in Biodiscovery Nexus Copy Number (El Segundo, CA).

## Conflict of interest

The authors declare no conflict of interests.

## Supporting information


**Table S1** SNP markers selected for the Wheat Breeders’ Array and associated information

**Table S2** Distribution of SNP loci exhibiting significant distortion of segregation

**Table S3** Genetic maps and consensus map data

**Table S4** Accessions assayed using the Wheat Breeders’ Array and genotype data (full data set available by accessing http://www.cerealsdb.uk.net/cerealgenomics/CerealsDB/Supplementary_file_3.xlsx)

**Table S5** Summary of copy number analysis for accessions screened using the Wheat Breeders’ Array
