# Supplementary material for: Characterization of a Wheat Breeders’ Array suitable for high‐throughput SNP genotyping of global accessions of hexaploid bread wheat (Triticum aestivum)
Source: Plant Biotechnol J. 2016 Nov 23;15(3):390–401. doi: 10.1111/pbi.12635 (PMC5316916; doi:10.1111/pbi.12635)
Supplement: Supplementary file 2 — Table S2 Distribution of SNP loci exhibiting significant distortion of segregation [file PBI-15-390-s005.docx]

| **Supplementary Table 2.** Distribution of SNP loci exhibiting significant distortion of segregation | | | | | |
| --- | --- | --- | --- | --- | --- |
| Cross | Chrom-osome | Position (cM) | No. significant SNPs (p<0.005) | Most significant p-value | Dominant genotype |
| Avalon x Cadenza | 1B | 12.2 | 1 | 0.00225 | Cadenza |
|  | 2A | 154.2, 186.0-187.2 | 12 | 1.12E-06 | Cadenza |
|  | 2D | 73.7 | 2 | 3.13E-06 | Avalon |
|  | 3B | 9.5-13.5 | 3 | 0.00245 | Cadenza |
|  | 4A | 42.1 | 1 | 0.00173 | Cadenza |
|  | 5B | 80.9, 116.0-130.4, 156.7-163.9 | 65 | 0.00044 | Cadenza |
|  | 6A | 78.7 | 1 | 0.00128 | Avalon |
|  | 7D | 179.0 | 1 | 0.00427 | Avalon |
| Savannah x Rialto | 3A | 71.5-74.7, 118.4 | 22 | 0.00096 | Rialto |
|  | 3B | 21.4-36.5 | 14 | 0.00067 | Savannah |
|  | 3B | 102.6 | 2 | 0.00115 | Rialto |
| Opata x Synthetic | None |  |  |  |  |
| Apogee x Paragon | 1A | 36.5 | 1 | 0.00024 | Paragon |
|  | 1B | 112.2, 145.0 | 2 | 0.00010 | Apogee |
|  | 2B | 46.2-106.7 | 6 | 0.00017 | Paragon |
|  | 2D | 1.8-9.26 | 2 | 0.00011 | Paragon |
|  | 2D | 136.0-269.0 | 17 | 5.17E-13 | Apogee |
|  | 3B | 200.2-217.6 | 15 | 5.38E-11 | Paragon |
|  | 5A | 79.7 | 1 | 0.00029 | Apogee |
|  | 6A | 221.3 | 1 | 2E-10 | Apogee |
|  | 6B | 54.9, 211.2 | 2 | 1.89E-06 | Apogee |
|  | 7A | 57.3-57.7 | 5 | 0.00017 | Paragon |
|  | 7B | 22.4, 234.5 | 2 | 0.00006 | Paragon |
| Chinese Spring x Paragon | 1A | 106.0-124.7 | 4 | 2.99E-06 | CS |
|  | 1B | 78.9, 200.3-254.8 | 4 | 0.00006 | CS |
|  | 1D | 7.98 | 1 | 0.00415 | CS |
|  | 2A | 171.4 | 1 | 0.00092 | Paragon |
|  | 2A | 341.1-368.8 | 23 | 3.21E-11 | CS |
|  | 2B | 27 | 1 | 0.00071 | CS |
|  | 2B | 103.1, 172.4, 232.2, 255.9 | 6 | 4.08E-05 | Paragon |
|  | 2D | 26.9, 160.0-219.0 | 97 | 2.38E-11 | CS |
|  | 3A | 9.5 | 1 | 0.00127 | CS |
|  | 3B | 7.6, 181.5, 260.0, 280.7 | 4 | 7.22E-07 | CS |
|  | 4A | 45.0, 118.5, 239.3 | 4 | 2.37E-06 | Paragon |
|  | 4A | 272.7 | 1 | 0.00014 | CS |
|  | 4B | 97.3 | 1 | 0.00115 | CS |
|  | 5A | 270.0 | 1 | 0.00033 | CS |
|  | 5A | 409.3-410.1 | 2 | 7.22E-05 | Paragon |
|  | 5B | 80.5, 202.9 | 2 | 0.00190 | Paragon |
|  | 5D | 27.7 | 1 | 0.00375 | CS |
|  | 6A | 2.6 | 1 | 0.00347 | CS |
|  | 6A | 287.8 | 1 | 0.00146 | Paragon |
|  | 6B | 71.1, 103.9-270.1 | 109 | 7.88E-10 | CS |
|  | 6D | 347.7-358.3 | 3 | 4.83E-07 | CS |
|  | 7A | 94.5-265.7 | 37 | 1.89E-08 | Paragon |
|  | 7B | 1.9, 4.3 | 3 | 0.00057 | Paragon |
|  | 7B | 35.4 | 1 | 0.00030 | CS |
|  | 7B | 62.5-69.5 | 2 | 3.46E-05 | Paragon |
|  | 7B | 72.1, 246.4-288.3 | 5 | 2.82E-06 | CS |
|  | 7D | 0.0 | 1 | 0.00120 | Paragon |
